# The effect of cassia seed extract on the regulation of the LKB1–AMPK–GLUT4 signaling pathway in the skeletal muscle of diabetic rats to improve the insulin sensitivity of the skeletal muscle

**DOI:** 10.1186/s13098-019-0504-0

**Published:** 2019-12-19

**Authors:** Qiu-Ying Wang, Ai-Hua Tong, Ying-Ying Pan, Xian-Dang Zhang, Wen-Yu Ding, Wen Xiong

**Affiliations:** 1 Endocrine Department, Heze Traditional Chinese Medicine Hospital, Heze, Shandong China; 2 Endocrinology Department, Linyi Central Hospital, Linyi, Shandong China; 3 Renal Rheumatism Immune Intervention Department, The People’s Hospital of Zhangqiu District, Jinan, Shandong China; 4 Endocrine Department, Shandong Institute of Endocrine & Metabolic Diseases, Shandong First Medical University & Shandong Academy of Medical Sciences, No. 18877, Jingshi Road, Lixia District, Jinan, 250062 China

**Keywords:** Cassia seed extract, Diabetes, Skeletal muscle, LKB1–AMPK–GLUT4, Insulin sensitivity

## Abstract

**Background:**

This study aimed to observe the hypoglycemic effect of cassia seed extract in rats with type-2 diabetes mellitus and its effect on reducing insulin resistance in the skeletal muscle.

**Methods:**

50 rats were randomly divided into the normal, model, high-dose, middle-dose, and low-dose groups of cassia seed extract (n = 10 each). A high-fat diet combined with streptozotocin administration was adopted to build type 2 diabetes models. The cassia seed extract groups were fed different concentrations cassia seed extract while the normal and model groups were fed the same volume of normal saline. The weight, FINS, GIR, insulin tolerance, blood glucose and blood lipid level, oxidative stress indices and expressions related to the LKB1–AMPK–GLUT4 pathway were detected and compared between the two groups.

**Results:**

Compared with the normal group, the model group showed lower weight, glucose infusion rate and expressions related to LKB1–AMPK–GLUT4 pathway and higher FINS, insulin tolerance, blood glucose and blood lipid level and oxidative stress indices (all P < 0.05). Compared with the model group, higher weight, glucose infusion rate and expressions related to LKB1–AMPK–GLUT4 pathway and lower FINS, insulin tolerance, blood glucose and blood lipid level and oxidative stress indices were observed in all groups that were administered cassia see extract (all P < 0.05).

**Conclusion:**

Cassia seed extract could noticeably improve the insulin resistance of diabetic rats and enhance the insulin sensitivity of their skeletal muscles. Its mechanism may be related to damage repair of the LKB1–AMPK–GLUT4 signaling pathway and oxidative stress in the skeletal muscle.

## Background

With continuous improvement in people’s quality of life in the recent years, the number of adult patients with diabetes mellitus has reached 0.285 billion and is showing a rising trend [1, 2]. As per the literature, the prevalence rate of diabetes is 9.7% among people aged > 20 years, and the total number of patients has exceeded 90 million in China, with type 2 diabetes becoming the most common endocrine and metabolic disease that threatens the health of the Chinese people. Studies have shown [3, 4] that insulin resistance (IR) in the peripheral tissues played a crucial role in the occurrence and development of type 2 diabetes, primarily manifesting as the barrier of glucose utilization by the muscular tissues, liver tissues, and adipose tissues. In order to compensate for the hypoglycemic effect, the pancreatic β cells need to increase the secretion of insulin. However, with disease progression, the pancreatic β cells gradually lose their function, resulting in abnormal glucose tolerance and type 2 diabetes. Therefore, treatment strategies to relieve the state of insulin resistance are being increasingly researched for the management of type 2 diabetes.

Researchers believe that skeletal muscle tissues are important targets for the occurrence of peripheral IR [5, 6], and the skeletal muscle IR in patients with type 2 diabetes can reduce the transport and uptake of glucose, directly leading to increased blood glucose levels. The LKB1–AMPK–GLUT4 signaling pathway is a vital signaling pathway for promoting the uptake, transport, and utilization of glucose in the skeletal muscle. Researches have shown [7, 8] that the LKB1–AMPK–GLUT4 signaling pathway could have abnormal expression in the skeletal muscle of patients with type 2 diabetes, consequently reducing the insulin sensitivity and increasing the blood glucose level [9, 10]. Hence, the activity regulation of the LKB1–AMPK–GLUT4 signaling pathway may be a novel research area for reducing insulin resistance and treating patients with diabetes.

Cassia seed is a frequently used traditional Chinese herb first documented in *Sheng Nong’s Herbal Classic* for its effects of liver detoxification, vision improvement, dispelling of the wind-evil, dissipating heat, and bowel relaxation. Modern pharmacological studies have indicated that the cassia seed had noticeable pharmacological effects, such as reduction in the blood lipid and blood pressure level as well as inhibition of lipid oxidation [11, 12]. Fu et al. [13] indicated that the cassia seed extract could effectively alleviate myocardial ischemia/reperfusion injury in diabetic rats and its mechanism may be related to the lipid-lowering effect and Akt and ERK1/2 signaling pathway activation. However, to the best of our knowledge, no report has yet proven whether the cassia seed extract reduces insulin resistance in the skeletal muscle. This study aimed to observe the hypoglycemic effect of cassia seed extract in rats with type 2 diabetes and its effect on reducing insulin resistance in the skeletal muscle. The report is shown in the following sections.

## Materials and methods

### Laboratory animals

A total of 50 male SD rats (SPF grade) aged 6 weeks, weighing 180–200 g were purchased from the Animal Experimental Center. Under the condition of alternating light and dark, all the rats were raised in separate cages of the animal house at a temperature of 22 °C ± 5 °C and humidity of 50% ± 10%. These rats were given ad libitum access to food and water, and were selected for this experiment after they were fed adaptively for 1 week.

### Laboratory reagents

Cassia seed extract was provided by the Nanjing Bangnuo Biotechnology Co., Ltd. Streptozotocin (STZ) was provided by Sigma Company. Rabbit P-LKB1, P-AMPKa12, AMPKa2, P-AMPKa2, and GLUT4 antibodies; anti-GAPDH rabbit antibody; protease inhibitor; and phosphatase inhibitor were provided by the American Sigma Company. The ECL kit was provided by Beijing Zhongshan Biotechnology Co., Ltd. The TaKaRa RNA PCR kit was provided by Takara Biotechnology (Dalian) Co., Ltd. The detection kit for glycogen in the skeletal muscle was provided by the Nanjing Jiancheng Bioengineering Institute, and the insulin kit was provided by Dalian Beyotime Biotechnology Institute.

## Methods

### Building and grouping of the animal model

A total of 10 rats were randomly selected and included in the normal group to be fed a normal diet. The other 40 rats were selected for the modeling of type 2 diabetes rats using the method described in the literature. The rats were fed a high-fat and high-sugar diet (fat 41%, protein 17%, carbohydrate 42%) for 8 weeks, followed by fasting of 12 h. Next, 35 mg/kg streptozotocin (STZ) was injected intraperitoneally once. Blood samples were collected from the caudal vein to detect the fasting blood glucose of rats 3 days after the injection. A fasting blood glucose level of ≥ 16.7 mmol/L indicated successful modeling of type 2 diabetes rats. These 40 rats that were modeled successfully were randomly divided into the model group, high-dose group of cassia seed extract, middle-dose group of cassia seed extract, and low-dose group of cassia seed extract.

### Administration method

The rats in the high-dose group, middle-dose group, and low-dose group were fed 450 150, and 50 mg/kg cassia seed extract, respectively, via intragastric administration once a day for 12 consecutive weeks. The rats in the normal group and model group were fed the same volume of normal saline via intragastric administration. After 12 weeks, these rats were killed for the assessment of the relevant indices.

### Determination of the weight, blood glucose, and fasting insulin

All the rats were continuously fed cassia seed extract for 12 weeks. Next, 24 h after the last administration and 12 h after fasting and water deprivation, 6 rats that died accidentally in the process of the experiment were removed, including 1 in the normal group, 2 in the model group, 1 in the high-dose group, 1 in the middle-dose group, and 1 in the low-dose group. After the death of the rats, the autopsy showed that the main reason for the death of the six rats was the damage to the esophagus during gavage, which led to severe pulmonary infection and the inability to eat, and eventually rats died of malnutrition. At the same time, the redundant rats in each group were removed randomly to keep eight rats in each group for the experiment. After weighing, the rats were intraperitoneally injected with 2% 40 mg/kg pelltobarbitalum natricum for anesthesia. Thereafter, blood samples were collected from the caudal vein to determine the fasting blood glucose and blood lipid levels. The insulin kit was used to detect the level of FINS. Lastly, the insulin sensitivity index (ISI) was calculated based on the method described in the literature.

### Measurement of the glucose infusion rate using the hyperinsulinemic–euglycemic clamp test

Three rats were selected for this experiment. After fasting and water feeding for 12 h, the rats were intraperitoneally injected with 2% Pelltobarbitalum Natricum for anesthesia. The rats were fixed to separate the left carotid artery and the right internal jugular vein, followed by the catheterization of PE-50 catheter in the blood vessel. Meanwhile, the insulin infusion and glucose infusion pumps were connected. After the basic insulin and basic blood glucose (BBG) were detected in the laboratory, the insulin was infused continuously at the speed of 0.12 U/(kg h). The blood glucose level was measured every 5 min, and 10% glucose was infused. Further, the infusion speed of glucose was adjusted as per the changes in the blood glucose level to maintain the level at (BBG ± 0.5) mmol/L. If the blood glucose was maintained at this level four times successively, it meant that the process of the clamp test was in a steady state. Under this condition, the time could be prolonged to 120 min, and the blood glucose level was measured for a total of 24 times in this experiment.

### Performance of the intraperitoneal insulin tolerance test

The rats were fed cassia seed extract for 4 weeks. Next, after fasting and water feeding for 12 h, the rats in each group were intraperitoneally injected with regular insulin and assessed respectively during the time periods of 0, 30, 60, and 120 min. Lastly, the area under the curve (AUC) was calculated.

### Determination of the oxidative stress level in the skeletal muscle tissues

The rats were intraperitoneally injected with 2% pelltobarbitalum natricum for anesthesia. After collecting the blood, the rats were sacrificed to obtain the gastrocnemius muscle rapidly that was then placed in a PE tube for homogenization. Next, the SOD, MDA, and ROS activities as well as the GSH level were detected according to the instructions on the kit.

### Detection of the protein expression related to the LKB1–AMPK–GLUT4 signaling pathway using the Western blotting method

The rats were killed to obtain 100-mg gastrocnemius muscle tissues. After quick-freezing in liquid nitrogen, 200-μL pre-cooling cell lysis buffer and corresponding phosphatase inhibitor were added for centrifugation at 12,000 rpm for 10 min at a temperature of 4 °C to extract the supernatant. The BCA method was then used to measure the total concentration of protein. After 100-μL protein and 100-μL sample buffer (2×) were taken out and boiled for 5 min for albuminous degeneration, the target protein was separated with SDS-PAGE electrophoresis. The next step was membrane transfer and sealing. Next, the corresponding primary antibody was added to perform TBST membrane rinsing 3 times, and the corresponding second antibody was added to conduct TBST membrane rinsing 3 times. Lastly, the BCIP enzyme chromogenic method was used for coloration, and the FlourChem V 2.0 gel-imaging analysis software (America) was used to analyze the gray value of the target band.

### Assessment of the GLUT4 mRNA expression using the real-time fluorescent quantitative PCR method

The method was the same as that in 2.6; 100-mg gastrocnemius muscle tissues were taken out; the Trizol method was used to obtain the total RNA in the gastrocnemius muscle. cDNA was synthesized according to the instructions of the ReverTra Ace qPCR reverse transcription kit. The GLUT4 mRNA primer was designed using the Primer Permier 5.0 software and synthesized by Sangon Biotech (Shanghai) Co., Ltd. The primer sequence is shown below: GLUT4: upstream sequence 5′-TGTTGCGGATGCTATGGG-3′ and downstream sequence 5′-CTGCGAGGAAAGGAGGGA-3′; and β-actin: upstream sequence: 5′-TCATCACTCGGCAATG-3′ and 5′-ACAGCACTGTGTGTTGGCAT-3′. The PCR reaction was amplified through AccuPower^®^ 2X Green StarqPCR Master Mix reaction system that contained SYBR GreenI. The conditions for PCR amplification included predegeneration of 95 °C for 10 s, degeneration of 95 °C for 15 s, and annealing of 55 °C for 20 s for total 40 cycles. Lastly, the 2^−ΔΔCt^ method was used to detect the relative expression quantity of the relevant genes in the gastrocnemius muscle tissues.

### Statistical analyses

The SPSS 21.0 statistical software package was used for data analysis. The measurement data are represented as mean ± standard deviation ($\bar{x} \pm s$) values. The two independent sample t-test was used to compare the means of two groups. Paired-samples t-test was used to compare the means in the same group before and after intervention. One-way ANOVA was used for the comparison of means between groups. P value < 0.05 indicates a statistically significant difference.

## Results

### Comparison of the weight, blood glucose, and FINS of all the groups

**Fig. 1:**
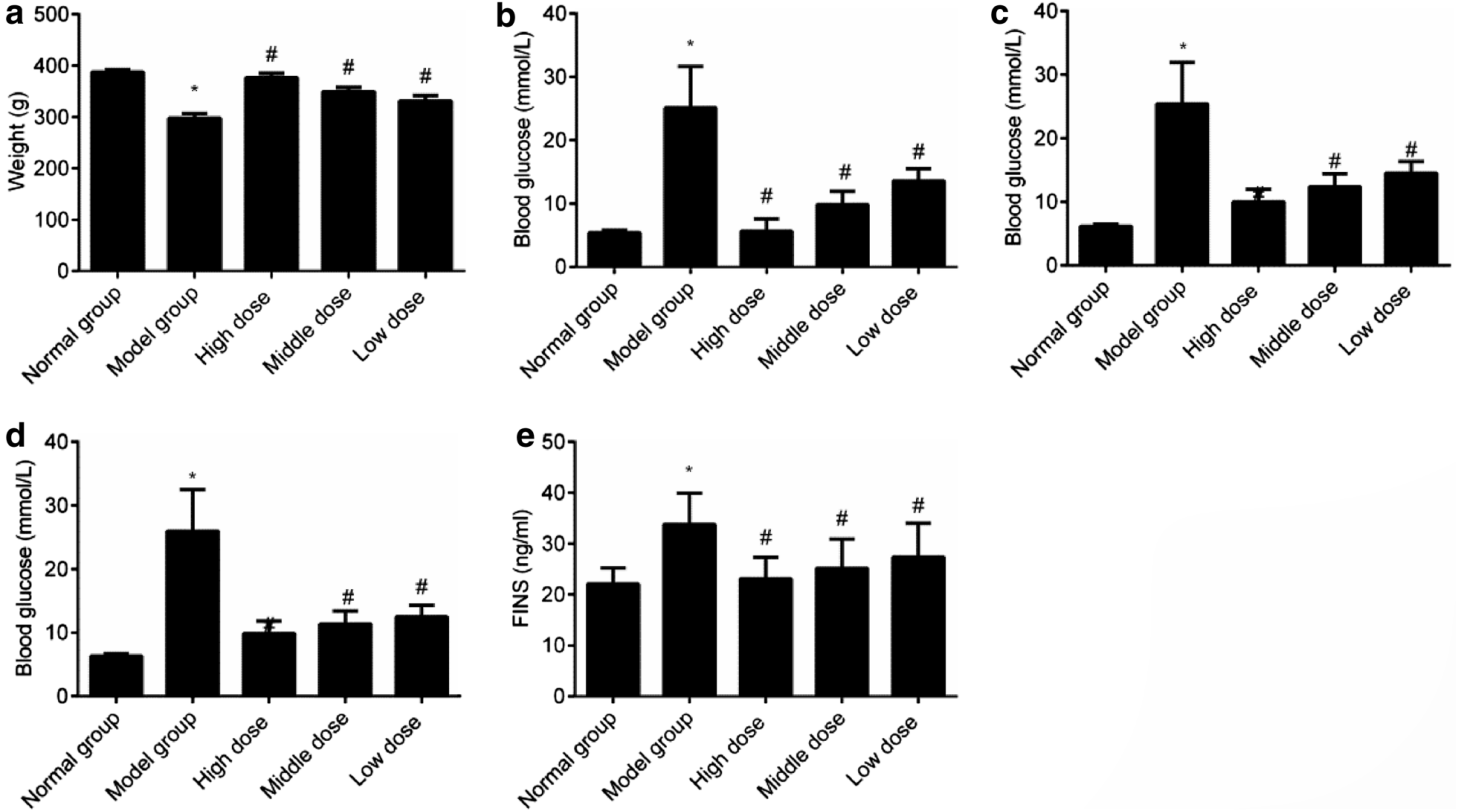
Comparison of the weight, blood glucose, and FINS among all the groups. **a** Weight; **b** Blood glucose; **c** 1h PG; **d** 2h PG; **e** FINS. *P < 0.05 indicates comparison with the normal group; and ^#^*P *< 0.05 indicates comparison with the model group

Compared with the normal group, the model group showed significant reduction in the weight and significant increase in the FBG, 1 hPG, 2 hPG, and FINS (*P *< 0.05). Compared with the weight in the model group, that in all groups administered cassia seed extract increased, and FBG, 1 hPG, 2 hPG, and FINS reduced in all groups administered cassia seed extract compared with that in the model group (*P *< 0.05). Dose dependence was noted in the comparison of all the groups administered cassia seed extract, implying that cassia seed extract can improve weight, blood glucose, and insulin level of diabetic rats, as shown in Fig. 1.

### Comparison of the GIR level of all the groups

**Fig. 2:**
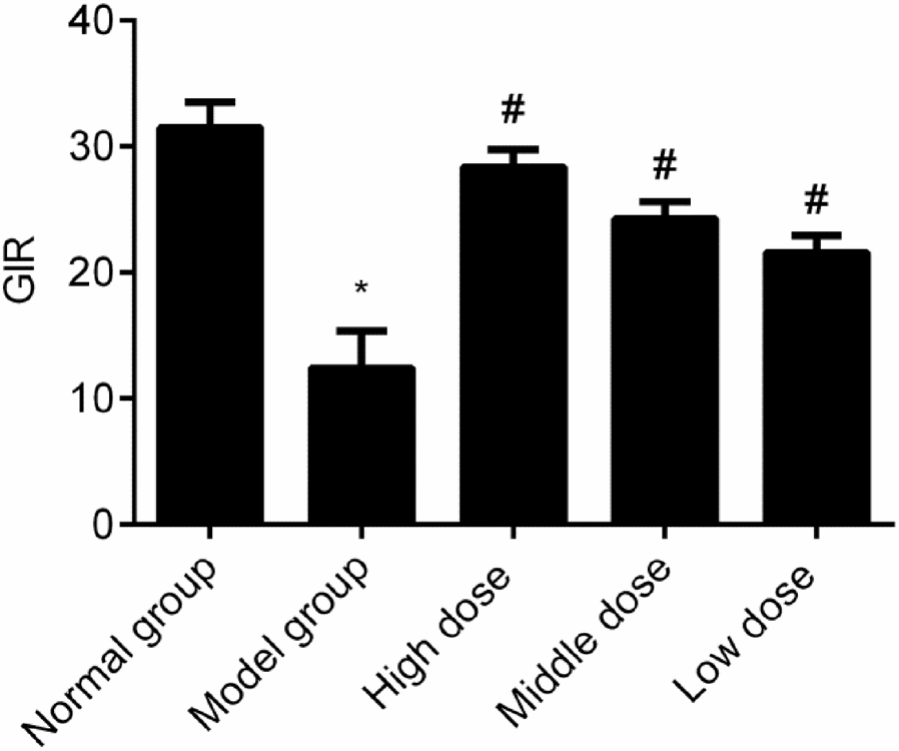
Comparison of the GIR level among all the groups. *P < 0.05 indicates comparison with the normal group; and ^#^*P *< 0.05 indicates comparison with the model group

The glucose infusion rate (GIR) of the model group reduced significantly (*P *< 0.05). Compared with that in the model group, the GIR level increased in all groups given the cassia seed extract (*P *< 0.05). There was a dose dependence in the comparison of all the cassia seed extract groups, indicating that cassia seed extract can improve the insulin sensitivity of diabetic rats, as shown in Fig. 2.

### Comparison of the insulin tolerance level of all the groups

The blood glucose level at all time points and the AUC increased in the model group (*P *< 0.05). Compared with that in the model group, the blood glucose level at all time points and the AUC reduced in all groups administered cassia seed extract (*P *< 0.05), as shown in Table 1.

**Table 1:** Comparison of the insulin tolerance level of all the groups (x ± s)

Group	n	0 min	30 min	60 min	120 min	AUC
Normal group	8	5.36 ± 0.62	3.41 ± 0.33	2.73 ± 0.31	3.14 ± 0.37	10.06 ± 0.60
Model group	8	13.97 ± 3.71*	11.97 ± 4.62*	8.36 ± 3.14*	8.48 ± 2.81*	32.41 ± 10.53*
High-dose group of cassia seed extract	8	5.57 ± 1.09^#^	3.81 ± 0.43^#^	3.22 ± 0.72^#^	4.02 ± 0.61^#^	11.10 ± 1.67^#^
Middle-dose group of cassia seed extract	8	5.69 ± 2.1.77^#^	4.22 ± 1.66^#^	3.88 ± 0.91^#^	3.76 ± 0.78^#^	14.24 ± 4.26^#^
Low-dose group of cassia seed extract	8	6.23 ± 2.05^#^	5.29 ± 1.76^#^	3.71 ± 0.89^#^	3.95 ± 0.83^#^	14.24 ± 4.26^#^

*P < 0.05 indicates comparison with the normal group; and ^#^*P *< 0.05 indicates comparison with the model group

### Comparison of the blood lipid level of all the groups

**Fig. 3:**
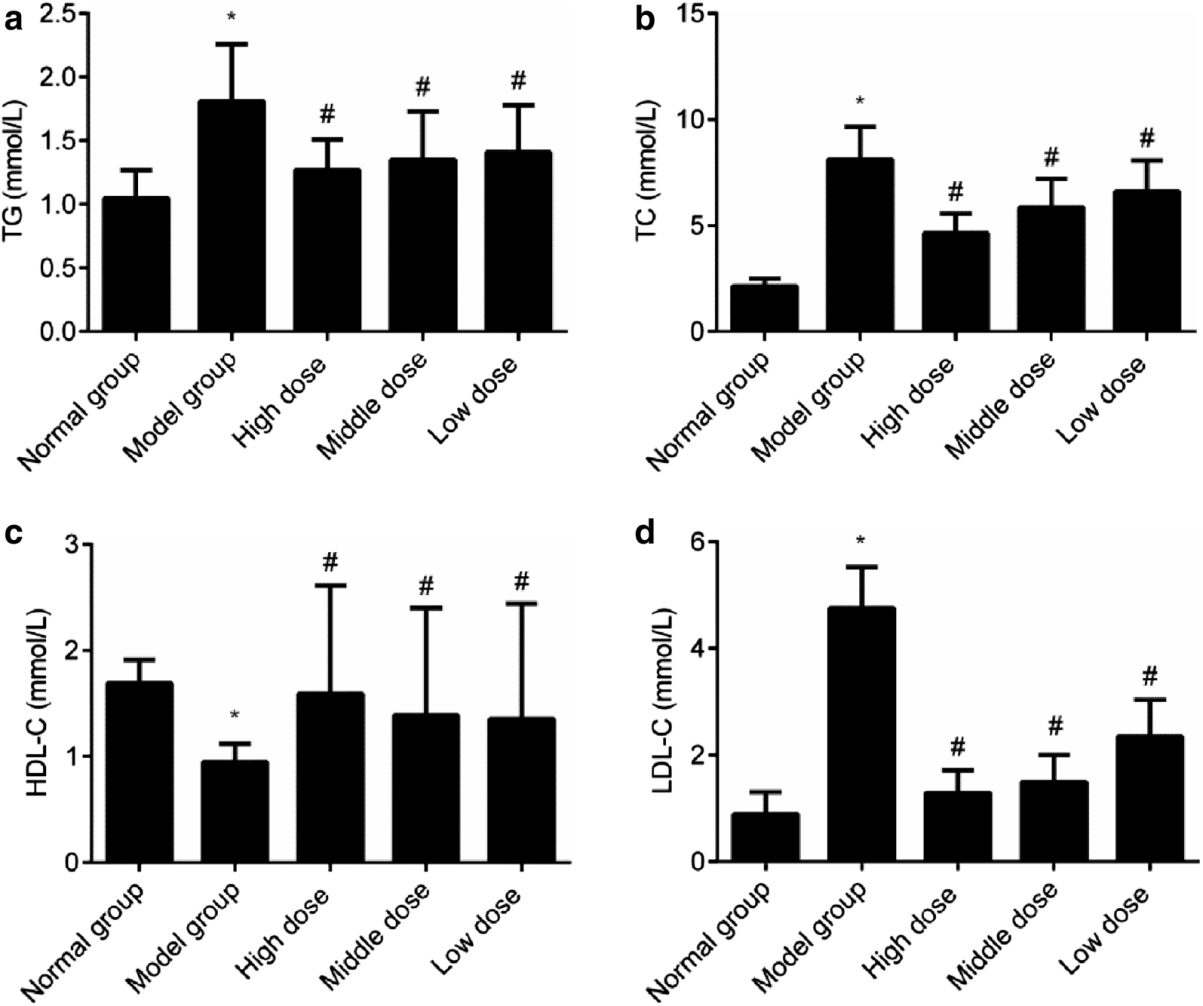
Comparison of the blood lipid level of all the groups. **a** TG; **b** TC; **c** HDL-C; **d** LDL-C. *P < 0.05 indicates comparison with the normal group; and ^#^*P *< 0.05 indicates comparison with the model group

TG, TC, and LDL-C in the serum increased significantly, while HDL-C reduced in the model group (*P *< 0.05). Compared with the model group, all groups given cassia seed extract showed significant reduction in the TG, TC, and LDL-C in the serum and significant increase in the HDL-C (*P *< 0.05). There was a dose dependence in the comparison of all the cassia seed extract groups, indicating that cassia seed extract could greatly improve the blood lipid level of diabetes rats, as shown in Fig. 3.

### Changes in the oxidative stress indices in the skeletal muscle among all the groups

**Fig. 4:**
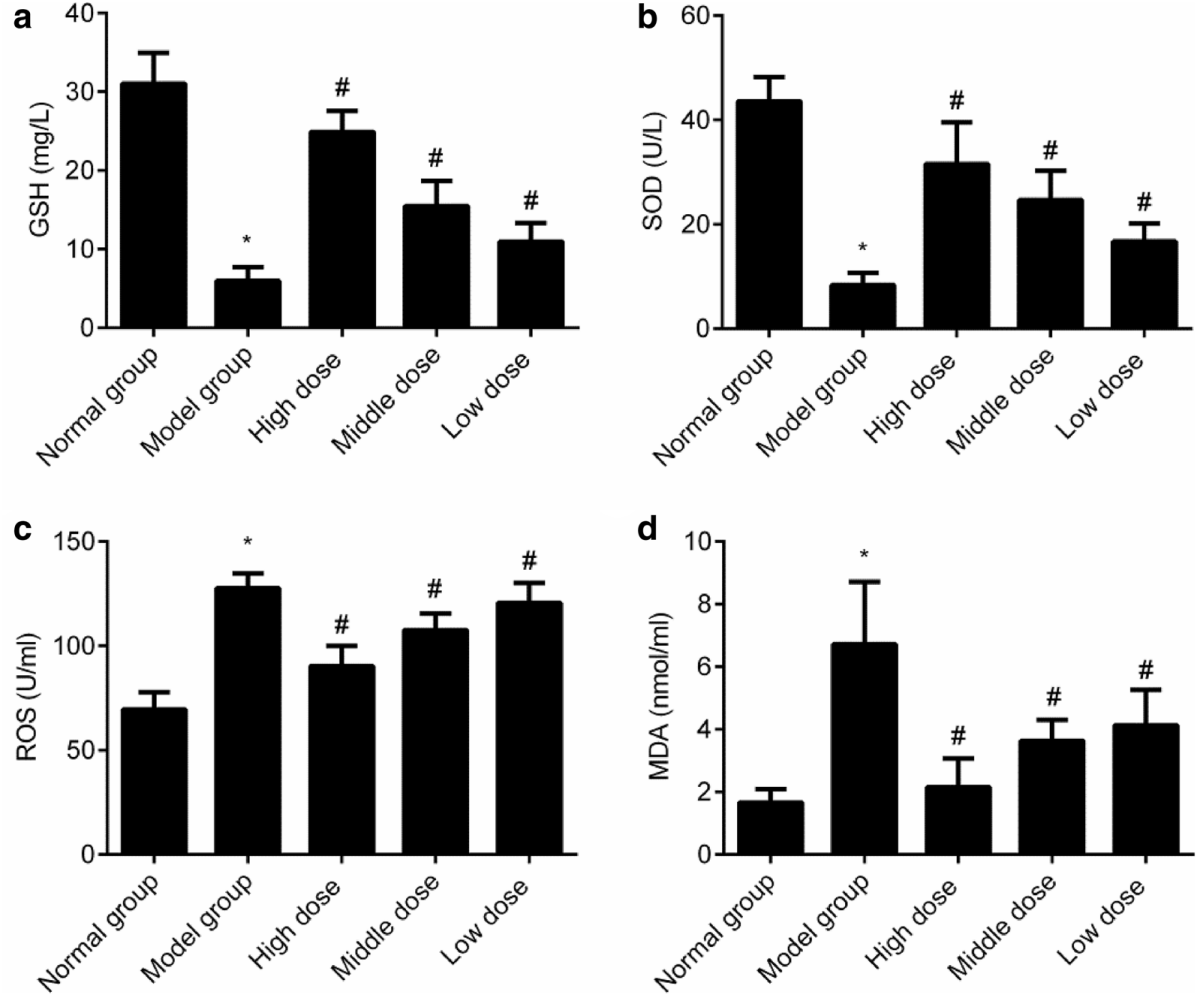
Comparison of the oxidative stress indices in the skeletal muscle of all the groups (x ± s). **a** GSH; **b** SOD; **c** ROS; **d** MDA. *P < 0.05 indicates comparison with the normal group; and ^#^*P *< 0.05 indicates comparison with the model group

The GSH level reduced and the SOD, ROS, and MDA levels increased in the model group (*P *< 0.05). Compared with that in the model group, the GSH level increased and the SOD, ROS, and MDA levels reduced in all groups given cassia seed extract (*P *< 0.05). This implied that cassia seed extract could greatly improve the oxidative stress level of diabetes rats, as shown in Fig. 4.

### Expression of the proteins related to the LKB1–AMPK–GLUT4 signaling pathway in the skeletal muscle among all the groups

**Fig. 5:**
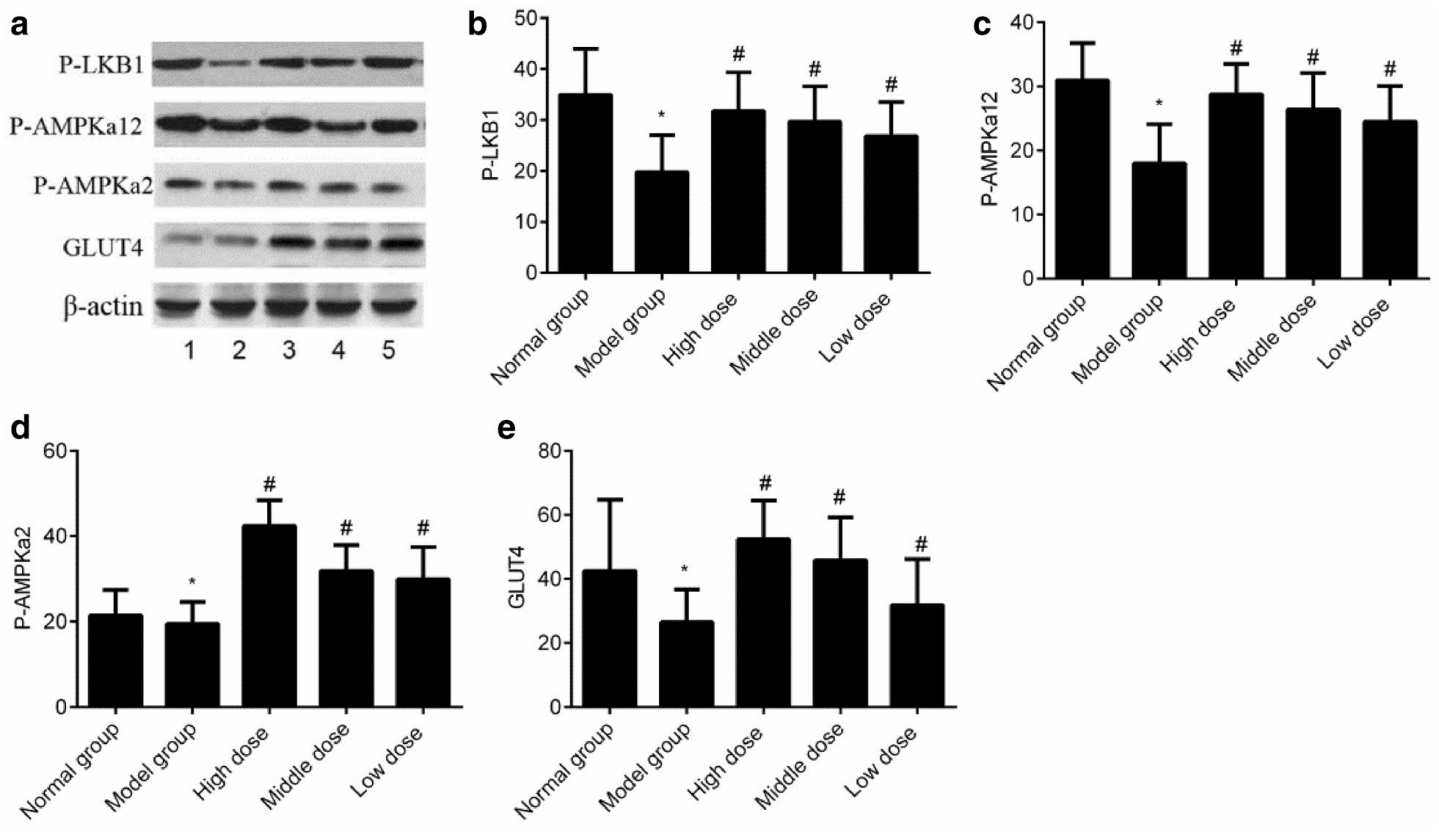
Expression of the proteins related to the LKB1–AMPK–GLUT4 signaling pathway in the skeletal muscle in all the groups. **a** Western blot of proteins related to the LKB1-AMPK- GLUT4; **b** Bar chart of protein relative expression of P-LKB1; **c** Bar chart of protein relative expression of P-AMPKa12; **d** Bar chart of protein relative expression of P-AMPKa2; **e** Bar chart of protein relative expression of GLUT4. *P < 0.05 indicates comparison with the normal group; and ^#^ *P *< 0.05 indicates comparison with the model group

The protein expression levels of P-LKB1, P-AMPKa12, AMPKa2, P-AMPKa2, and GLUT4 were reduced in the model group (*P *< 0.05). Compared with that in the model group, the protein expression levels of P-LKB1, P-AMPKa12, AMPKa2, P-AMPKa2, and GLUT4 were increased in all groups given cassia seed extract (*P *< 0.05), as shown in Fig. 5.

### Expression of the genes related to the LKB1–AMPK–GLUT4 signaling pathway in the skeletal muscle among all the groups

**Fig. 6:**
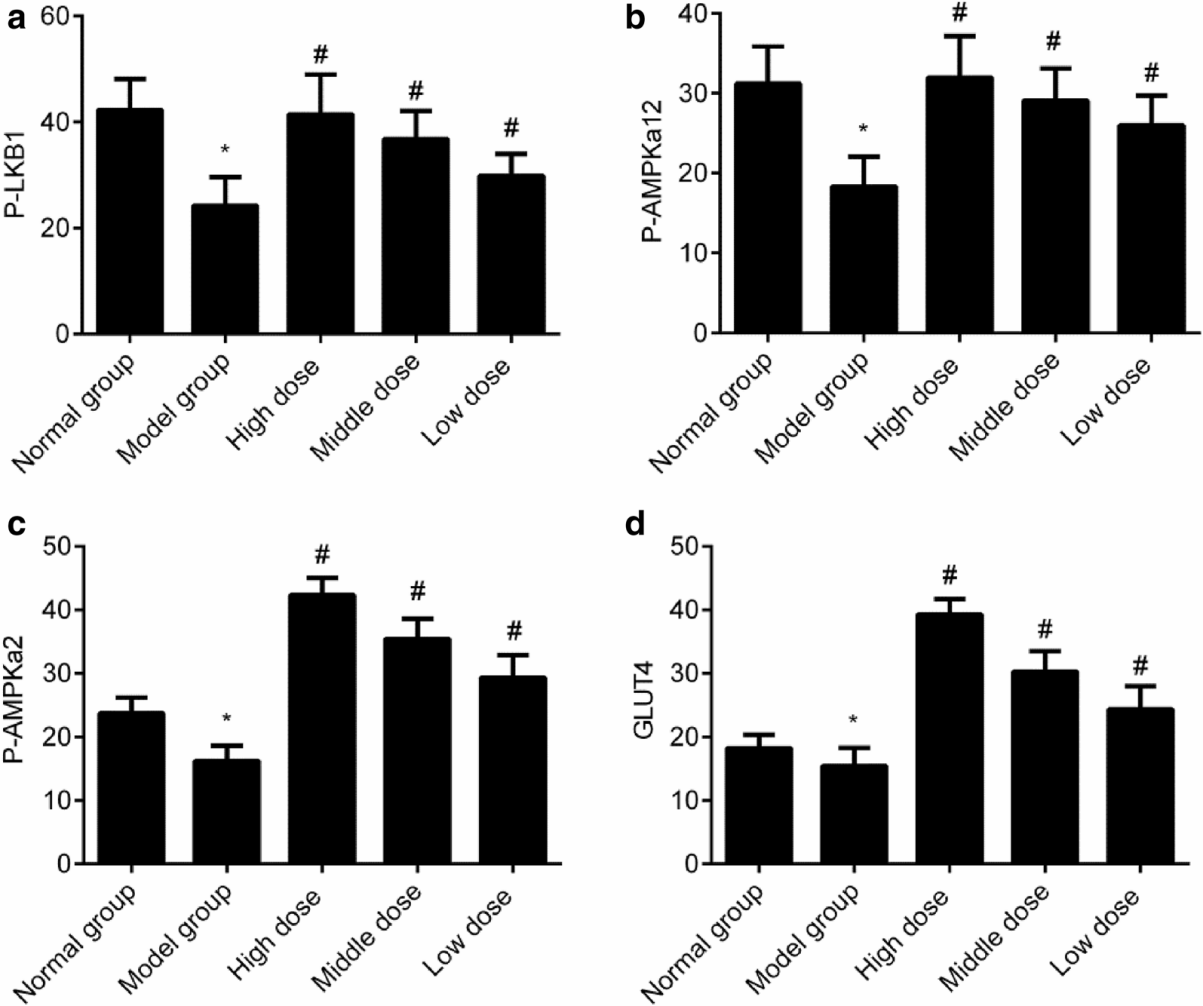
Expression of the genes related to the LKB1–AMPK–GLUT4 signaling pathway in the skeletal muscle in all the groups. **a** P-LKB1 mRNA; **b** P-AMPKa12 mRNA; **c** P-AMPKa2; **d** GLUT4 mRNA. *P < 0.05 indicates comparison with the normal group; and ^#^*P *< 0.05 indicates comparison with the model group

The expression levels of P-LKB1, P-AMPKa12, P-AMPKa2, and GLUT4 mRNA were reduced in the model group (*P *< 0.05). Compared with that in the model group, the expression levels of P-LKB1, P-AMPKa12, P-AMPKa2, and GLUT4 mRNA were increased in all groups given cassia seed extract (*P *< 0.05), as shown in Fig. 6.

## Discussion

Insulin resistance is a crucial pathophysiologic foundation in the pathogenetic process of type 2 diabetes. Therefore, the primary place for insulin to stimulate glucose uptake is the skeletal muscle, making the skeletal muscle the key part that leads to the insulin resistance in the peripheral tissues [8, 14]. AMPK is one of the members of the serine-threonine kinase family. In a normal body, the phosphorylation of upstream tumor-inhibiting factor-1 (LKB1) that is activated can phosphorylate the Thr172 locus on AMPKa subunit and thus activate AMPK that will promote the transport process of glucose transporter-4 (GLUT4) to glucose and the processes of glucose uptake, transport, and utilization in skeletal muscle and improve the insulin resistance level of patients. Meanwhile, this can also promote the metabolism of triglycerides and free fatty acid in the blood and regulate the uptake and utilization of glucose in the skeletal muscle [15, 16]. Some researches have shown [17] that the inhibition of the expression of the LKB1–AMPK–GLUT4 signaling cascade in the skeletal muscle of patients with type 2 diabetes reduced the transfer efficiency of glycogen in the skeletal muscle, thus decreasing the insulin sensitivity and glucose disposal rate in the blood and increasing the blood glucose level. Therefore, the LKB1–AMPK–GLUT4 signaling pathway is a vital signaling pathway for the metabolic pathway of diabetes.

In addition, some other researches have shown [18, 19] that oxidative stress injury existed in the overall development process of type 2 diabetes. The increase in blood glucose and fatty acid can induce the body to generate a large amount of ROS [19, 20], enhance the insulin resistance of the skeletal muscle, promote the recession of β cell function, increase the tolerance level of glucose, and thus cause and aggravate the condition of patients with type 2 diabetes. Thus, GSH and SOD play an important role of reducing agents in cell oxidation reaction to promote the removal of oxidative metabolites and reduce the incidence of lipid peroxidation reaction [21, 22]. MDA and ROS are vital markers that reflect the generation of oxygen radicals and the damage of tissues in the process of oxidative stress reaction [23, 24].

In this study, the model of type 2 diabetes rats was prepared successfully, and the results that compared with that in the normal group, the weight and GIR level reduced significantly, while the FBG, FINS, and AUC increased significantly in the model group. This indicated the successful modeling of type 2 diabetes rats and the occurrence of insulin resistance. At the same time, in order to further confirm the hypoglycemic effect of cassia seed extract, we administered different doses of cassia seed extract to diabetic rats. In order to avoid the effect of injecting fluid on blood glucose fluctuations during administration, cassia seed extract was configured with normal saline. The normal group and the model group were given the same amount of normal saline to ensure the consistency of caloric output between the groups and avoid the blood glucose fluctuations. Furthermore, all the indices had improved significantly after the administration of cassia seed extract; thus, cassia seed extract can considerably reduce the hyperglycemic state and improve insulin resistance in rats with type 2 diabetes.

Moreover, the results also indicated that the expression levels of P-LKB1, P-AMPKa12, P-AMPKa2, GLUT4 mRNA, and proteins reduced significantly in the model group, showing that the LKB1–AMPK–GLUT4 signaling cascade was not only inhibited markedly under the pathological condition of diabetes, but also contributed to the development of diabetes. At the same time, the GSH level reduced significantly, while the SOD, ROS, and MDA levels increased significantly in the model group, indicating that the oxidative stress level increased significantly in type 2 diabetes rats and the oxidative stress may have contributed to the occurrence of type 2 diabetes. After the treatment with cassia seed extract, the expression levels of P-LKB1, P-AMPKa12, AMPKa2, P-AMPKa2, GLUT4 mRNA, and proteins increased significantly; the GSH level increased significantly; and the SOD, ROS, and MDA levels reduced significantly in all groups that were administered cassia seed extract, indicating that cassia seed extract could significantly reduce the level of oxidative stress level in type 2 diabetes rats, alleviate the damage to the LKB1–AMPK–GLUT4 signaling pathway in the skeletal muscle, enhance the insulin sensitivity, and delay the disease condition in type 2 diabetes.

## Conclusions

In conclusion, cassia seed extract could obviously improve the insulin resistance of diabetes rats and enhance the insulin sensitivity of the skeletal muscle. Its mechanism may be related to the repair of damage to the LKB1–AMPK–GLUT4 signaling pathway in the skeletal muscle and reduced damage due to oxidative stress. However, this study failed to include the correlation analysis between the LKB1–AMPK–GLUT4 signaling pathway and oxidative stress reaction; this subject needs to be researched in further studies. At the same time, this is a preliminary study, and the indicators of observation are not detailed enough to comprehensively observe the relevant pathway factors from multiple perspectives. In the next study, immunohistochemistry and immunofluorescence will be added to further activate the inflammatory pathways.

## Data Availability

Not applicable.

## Abbreviations

STZ: streptozotocin
HEC: hyperinsulinemic–euglycemic clamp
IPITT: intraperitoneal insulin tolerance test
FINS: fasting insulin
GIR: glucose infusion rate
AUC: area under the curve
IR: insulin resistance
ISI: insulin sensitivity index
BBG: basic blood glucose
LKB1: phosphorylation of upstream tumor-inhibiting factor-1
GLUT4: glucose transporter-4
